# Unveiling the In Vitro Antibiofilm Efficacy of Antifungal Lipopeptides Purified from *Bacillus* sp. against Mixed-Species Biofilms of *Candida*

**DOI:** 10.1021/acsomega.5c06938

**Published:** 2025-11-21

**Authors:** Madhuri Madduri, Shivaprakash M. Rudramurthy, Utpal Roy

**Affiliations:** † Department of Biological Sciences, BITS Pilani K.K. Birla Goa Campus, NH 17B Bypass Road, Goa 403726, India; ‡ Department of Medical Microbiology, 29751 Post Graduate Institute of Medical Education and Research (PGIMER), Chandigarh 160012, India

## Abstract

Polymicrobial fungal infections are often associated with significant invasive mycosis. *Candida* non-*albicans* species such as *Candida glabrata* and *Candida tropicalis* are most frequent organisms after *Candida albicans* especially in biofilm-associated fungal infections with significant mortality and morbidity. In the present investigation, the antibiofilm efficacy of *Bacillus*-derived lipopeptides AF_4_ and AF_5_ was assessed against *Candida* dual species mixed biofilms at different stages such as adherence, developmental, and mature phase in vitro experiments. Interestingly, in this study, *C. glabrata* was found to be predominant over *C. tropicalis*, whereas AF_4_ and AF_5_ exposures significantly reduced both biofilm biomass and metabolic activity as was revealed through various experiments such a quantification assays and morphological analyses. The advanced microscopy suggests the strong biofilm-busting activity of both lipopeptides. Additionally, the elucidation of their mode of action against the planktonic phase of *C. glabrata* and *C. tropicalis* was performed to investigate the presence of stages of apoptosis with Annexin V-FITC/propidium iodide staining and TUNEL assays. Two strains were exposed to sub-MIC and minimum inhibitory concentration (MIC), also with 2× MIC of AF_4_ and AF_5_. A significant shift from live cells to early stage and late-stage apoptotic induction percentage was increased along with increasing the concentration of 1× MIC to 2× MIC. TUNEL results confirmed the DNA fragmentation by the enhanced green fluorescence intensity in cells in TUNEL-positive nuclei. Taken together, our results demonstrate that *Bacillus*-derived lipopeptides AF_4_ and AF_5_ hold great promise with strong antibiofilm properties. The overall antibiofilm properties and mode of action characterization studies unequivocally confirm the antifungal potential of the two investigational lipopeptides, with the ability to challenge several fungal infections.

## Graphical abstract

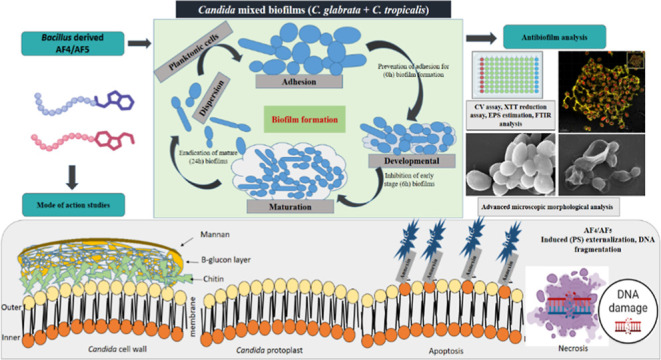

## 1 Introduction

*Candida glabrata* and *Candida tropicalis* are listed as high prioritized fungal pathogens according to World Health Organization 2022.^1^ Candidiasis, a fungal infection, causes both superficial and systemic mycosis with high morbidity and mortality.^2^ *Candida* species are human commensal fungal organisms, very well-known for causing opportunistic fungal infections in immunity compromised hosts especially hospitalized patients.^3^ Non-*albicans Candida* species such as *C. tropicalis*, *C. glabrata*, and *Candida parapsilosis* most commonly coisolated fungal pathogens after *Candida albicans* *.* According to the fungal nomenclature, *C. glabrata* is currently known as *Nakaseomyces glabratus* or *Nakaseomyces glabrata* and previously known as *Torulopsis glabrata*.^4,5^ *C. glabrata* can potentially disseminate throughout the host system^6^ and can cause infection with a significant mortality rate. Besides, *C. glabrata* is endowed with the uncanny ability to adhere and to develop biofilms on host tissues which render the infections clinically challenging since the cells possessed by biofilms happen to be intrinsically resistant to several antifungal treatments.^7^ The environmental adaptability of these species increases the adherence to the biotic and abiotic surfaces, and forming colonization is one of the major virulent factors associated with biofilm infections in humans.^8–10^ *Candida* biofilms possess a significant challenge in the realm of human health, specifically due to their relation to persistent infections in immunocompromised individuals.^1,11,12^

Biofilm formation through two different interspecies (fungi/bacteria) interacting and exchanging secreted substrates benefits the microorganism with enhanced antimicrobial resistance forms of persistence.^13,14^ While many microorganisms can form single-species biofilms, it is far more common to encounter polymicrobial biofilms, consisting of two or more bacterial and fungal species.^13,15,16^ These polymicrobial biofilms often confer specific advantages to each species compared to single-species biofilms.^10,17^ Indeed, biofilms are a protected niche for microorganisms, as well as cells within the biofilms possess properties distinct from their planktonic (free-floating) counterparts, which exhibits more resistance to drugs and physical disruptions. Distinguish between planktonic and multicellular organisms, the shift between these stages is critical for biofilm development.^11,18^ It involves the transition from planktonic to sessile cells for biofilm formation, as well as the detachment of sessile cells, which allows for a return to the planktonic phase. Sessile cells inside biofilms usually have a sedentary or inactive development phase.^19,20^ In particular, mature biofilms exhibit various phenomena influenced by fungal characteristics and morphological transitions, which can also play an integral role in host–pathogen interactions.^12,21^ In our previous study, the lipopeptides produced by *Bacillus subtilis* demonstrated a significant antibiofilm activity against a monospecies *C. glabrata* biofilms^22^ and *C. tropicalis* biofilms;^23^ the AF_4_ and AF_5_ lipopeptides previously demonstrated a wide-spectrum antifungal activity (by AFST according to CLSI guidelines) that inhibited about 120+ isolates/strains of *C. albicans*, non-*albicans Candida* (including *C. glabrata*, *C. tropicalis*, and *Candida auris*) and filamentous species.^24,25^ The present investigation was aimed at studying the effects of two antifungal lipopeptides isolated from the producer strain *B. subtilis* on the *Candida* mixed-species biofilms at different stages, and additionally, lipopeptides were investigated to determine their effects on the fungal cell death and DNA, as described in the graphical abstract.

## 2 Materials and Methods

### 2.1 Production and Purification of Antifungal Lipopeptides AF_4_ and AF_5_ through Preparative Scale HPLC

Briefly, AF_4_ and AF_5_ antifungal lipopeptides are isolated from *B. subtilis* RLID 12.1 using the multistep purification process as described in.^26^ 60 h grown *Bacillus* cells secreted secondary metabolites were collected in the form of the supernatant subjected to acid precipitation followed by organic solvent extraction further partially purified by adsorption column to isolate the active molecules with spot-on-lawn assay against *Candida* species on the freshly prepared Sabouraud dextrose agar (SDA). Preparative scale-HPLC (Shimadzu Models: LC2010C HT, Prominence-i LC2030C Plus, and i-Series LC2050C) with a reverse phase C18 column (30 × 250 mm (HSS)) with 10 μm porosity to purify the antifungal lipopeptides homologues from the active fractions was described in.^23^ The bicinchoninic acid method was employed to quantify the antifungal lipopeptides concentrations after the purification to assess their antibiofilm or mode of action studies, as illustrated in the graphical abstract and elsewhere.

### 2.2 *Candida* Strains and Culture

*C. glabrata* American Type Culture Collection (ATCC) 2001 and *C. tropicalis* ATCC 750 were maintained as the glycerol stocks at −80 °C and revived on the SDA plate. On Hi-CHROMagar *Candida* (Chromagar, HiMedia, Mumbai), a differential agar medium was used to grow *C. glabrata* and *C. tropicalis*. The fungal organisms on chromogenic differential media appeared as creamy white to pinkish colonies for *C. glabrata* and as bluish colonies for *C. tropicalis*. The RPMI-1640 containing l-glutamine, phenol red, 0.2% glucose, and 0.165 M MOPS (morpholinepropanesulfonic acid) (pH 7.0 ± 0.2) medium without sodium bicarbonate was used for the experiments.

### 2.3 Determination of Biofilm Formation

For biofilm formation assessment, a single colony from 24 h subcultured plates of each strain *C. glabrata* and *C. tropicalis* was picked and inoculated separately into 10 mL of Sabouraud dextrose broth and incubated for 18 h at 37 °C with 120 rpm. To prepare the inoculum for biofilm formation, cells were harvested by centrifugation at 3000*g* for 10 min at 4 °C and washed twice with sterile phosphate buffer saline (PBS).^22^ A volume of 100 μL of yeast cell suspension (10^6^ cells/mL) adjusted in RPMI-1640 with 0.2% glucose, from each strain, was added to 96-well polystyrene (PS) plates.

### 2.4 Prevention of Adhesion and Inhibition or Eradication Effect on Developmental and Mature Phase Biofilms with AF_4_ and AF_5_

To analyze the effect of lipopeptides (AF_4_ or AF_5_) alone or in combination with fluconazole (FLC) for prevention of adhesion in the biofilm formation, log phase cells were added along with the drug into the 96-well plate. At the early phase of biofilm formation (0 h), 100 μL of a standardized cell suspension (10^6^ CFU/ml) of each strain (*C. glabrata* and *C. tropicalis*) in (1:1 equal portion) was added to RPMI-1640 with 0.2% glucose medium containing (AF_4_ or AF_5_) alone or with FLC for evaluating the synergetic effect. Compounds were added to each well except the media (drug-free) control wells and growth control wells, and plates were incubated at 37 °C for 24 h at 75 rpm under shaking conditions. Further biomass quantification and metabolic activity, along with surface morphological analysis techniques, were performed.

To evaluate the inhibition of the developmental stage or eradication effect on matured biofilm, 200 μL of each of *C. tropicalis* and *C. glabrata* adjusted (10^6^ CFU/mL) of mixed cell suspension in a 1:1 ratio was added to each well. Plates were incubated for 6 h for developmental stage evaluation and for the mature stage, cells were incubated for 24 h. Postincubation, preformed mixed-species biofilms were washed with 1xPBS. Then, the lipopeptides (AF_4_ or AF_5_) were added in the presence of RPMI-1640 and further incubated for an additional 24 h.

### 2.5 CV and XTT Reduction Assays

For biofilm formation, presterilized, PS, flat-bottomed, commercially available 96-well microtiter plates were used, and the quantifications of biofilm biomass and metabolically active cells were determined after treatment with antifungal lipopeptides of AF_4_ and AF_5_ (8 and 16 μg/mL) and FLC (32, 64, and 128 μg/mL) alone and in combination with these concentrations.

Protocols for the XTT dye reduction and CV assays have been described.^22,27,28^ Post-treatment, biofilms were fixed with 100 μL of 99% methanol, and 1% (v/v) CV in 25% methanol aqueous CV was added for 20 min and washed with autoclaved double distilled water. 200 μL of 33% acetic acid (v/v) in water was added to release bound CV; the absorbance measurement (590 nm) was taken from each well. For the XTT reduction assay, 200 μL of XTT solution containing menadione was added to the reaction to measure the metabolically active cells in the biofilm, further incubated in the dark for 3 h at 37 °C, and at 492 nm, the absorbance was recorded using a microplate reader. Reproducibility was estimated by the standard deviation and the coefficient of variation among replicates. At least three independent experiments in triplicate were performed for each experiment.

### 2.6 EPS Estimation and FTIR Analysis

The method described in^29^ was followed to measure the extracellular polysaccharides in both untreated and treated samples of the *C. glabrata* biofilm. Briefly, mature biofilms were grown in a 24-well plate for 24 h, following which the drug was added to the wells. After 24 h of incubation with the respective drug, cells were washed with sterile 0.9% saline and transferred to sterile test tubes, and an equal volume of 5% phenol and five volumes of concentrated sulfuric acid were added to the respective tubes containing cell suspension. It was followed by 1 h of dark incubation, and subsequently, the absorbance was recorded at 490 nm.

FTIR spectroscopy potentially identifies the presence or abundance of particular chemical functional groups within the biofilms, such as those in the amide region (related to proteins) and those containing phosphates (found in nucleic acids and some lipid moieties). Additionally, FTIR enables the detection of functional groups in the composition of the EPS, the matrix that holds the biofilm together, including polysaccharides, proteins, and other molecules. For the FTIR analysis,^30^ biofilms were transferred from the 24-well plate into centrifuge tubes after washing with sterile 0.9% saline, then samples were mixed thoroughly and centrifuged for 15 min at a cold temperature at 3000*g*. The resulting cell pellets were directly placed in contact with the diamond crystal of the PerkinElmer Spectrum two FTIR spectrometer ATR-FTIR. Biofilm analyses were carried out within the wavenumber range between 3000 cm^–1^ and 500 cm^–1^, at a resolution of 4 cm^–1^. Each final spectrum represented an average of 64 scans.

### 2.7 Biofilm Morphological Analysis Using Advanced Microscopy

For the morphological visualization and analysis the biofilm cells of mixed species treated with AF_4_ and AF_5_, scanning electron microscopy (SEM) and confocal scanning laser microscopy (CSLM)^31,32^ were used. In brief, the biofilms were grown on PS coverslips that were placed in a 24-well culture plate; the coverslips were autoclaved, then surface sterilized using 70% ethanol, and air-dried before being placed in the sterile 24-well plate. The 200 μL of a standardized (10^6^ CFU/mL) cell suspension of each strain in RPMI-1640 medium was added to the 24-well plate. After (6 or 24 h), AF_4_ or AF_5_ was added at 8 and 16 μg/mL concentrations, and the plates were incubated further for 24 h.

#### 2.7.1 Scanning Electronic Microscope (SEM) Analysis of Biofilm Architecture

Post-treatment, after incubation, samples were washed with 1X PBS and processed for SEM analysis.^27^ In brief, the biofilms were washed twice with sterile PBS. Next, the biological samples were fixed with 2.5% glutaraldehyde (1 h at room temperature), and 0.1 M sodium cacodylate buffer was used to remove the glutaraldehyde by washing twice, followed by osmium tetroxide treatment for 30 min to improve the conductivity, and again allowed a wash with 0.1 M sodium cacodylate buffer. The dehydration process was followed with a graded series of 30% to 100% ethanol. The dehydrated samples were fixed on aluminum stubs, sputter coated with gold, and observed using the SEM (FEI, Quanta 250 FEG 30 kV) at different magnifications.

#### 2.7.2 Confocal Microscope (CSLM) Biofilm Observation

For the confocal analysis, post- (lipopeptide)-treated biofilms were washed twice with 1X PBS, and incubation was carried out in the presence of sterile buffer containing the fluorescent stain FUN-1 and Concanavalin A (Con A)–Alexa Fluor 488 conjugate that were used after dilution to appropriate concentrations from the respective stocks. Images were captured using an inverted confocal microscope system (Olympus FV3000, Central Sophisticated Instrumentation Facility, BITS Goa).

### 2.8 Apoptosis

#### 2.8.1 Protoplast Preparation

In order to determine the physiology and mode of cell death in response to two individual antifungal lipopeptides, AF_4_ at 2, 4, and 8 μg/mL and AF_5_ at 4, 8, and 16 μg/mL for 12 h against *C. glabrata* and *C. tropicalis,* expression of apoptotic markers was analyzed by spotting transfer of phosphatidylserine (PS) from inside to outside of the cell membrane as previously described by.^33,34^ Briefly, cell protoplasts were prepared by digesting the yeast cell wall with zymolyase (US Biologicals, USA) in different washing steps in protoplast buffers (pH 7.4). Prior cells were washed twice with protoplast buffer containing 1 M sorbitol, 50 mM Tris base, and 10 mM MgCl_2_, and dithiothreitol (DTT) (30 mM) was incubated for 15 min at 25 °C. After centrifugation at 1500 rpm for 5 min, the supernatant was removed and incubated with lyticase enzyme (≥1 μg/mL) for 60 min at 25 °C in the same buffer with 1 mM DTT. Followed by centrifugation at 1500 rpm for 5 min, cells were washed with the same buffer without DTT and incubated for 20 min at room temperature. Finally, protoplasts were washed with PBS and dissolved in binding buffer for further use.

#### 2.8.2 Annexin V-FITC/PI Staining

The experiment was performed by using an Annexin V- FITC apoptosis detection kit (MedChem Express, USA) following the instruction manual. Induction of apoptosis was assessed by using an Annexin V-FITC/PI kit. As previously mentioned, the treated *C. glabrata* and *C. tropicalis* protoplast cells (0.5 McFarland) were suspended in 100 μL of 1× binding buffer (available in the kit).^35^ The protoplasts were stained with Annexin V-FITC and PI, according to the manufacturer’s instructions. Thereafter, samples were analyzed using a FACScan flow cytometer (Becton Dickinson FACS Melody), and results were analyzed using FlowJo_V10 software. Untreated protoplasts were included as negative and also H_2_O_2_-treated protoplasts were considered as a positive control. Cell populations were divided into four different quadrants with Quadrants 1: necrosis (Annexin V^–^/PI^+^); Quadrants 2: late apoptosis (Annexin V^+^/PI^+^); Quadrants 3: live cells (Annexin V^−^/PI^−^) and Quadrants 4: early apoptosis (Annexin V^+^/PI^−^).

#### 2.8.3 TUNEL Assay

TUNEL assay was performed to detect DNA damage, a key indicator of apoptosis, wherein the Click-iT Plus TUNEL assay kit was in accordance with the manufacturer’s instruction protocol. Planktonic *Candida* cells treated with the lipopeptide AF_4_ (4 and 8 μg/mL) were fixed with 4% paraformaldehyde and incubated at room temperature for 15 min. Protoplasts were prepared as previously described.^33,36^

Protoplast permeabilization was achieved using 0.25% Triton X-100 in PBS followed by rinsing twice with deionized water. TdT reaction buffer was added to each sample and incubated for 10 min at 37 °C. After removing the buffer, the TdT reaction mixture was added, and the samples were incubated for an additional hour at 37 °C in a humidified chamber. The protoplasts were washed twice with 3% bovine serum albumin (BSA) in PBS for 5 min each, followed by the immediate addition of the Click-iT Plus TUNEL reaction cocktail to each sample. We incubated the samples at 37 °C in the dark for 30 min and then washed them once with 3% BSA in PBS. The cells were counterstained with Hoechst 33342 dye in the dark at room temperature. Following this, we washed the samples twice with PBS. Images were captured using an inverted CSLM (Olympus FV3000, in-house facility) at excitation and emission wavelengths of 495 and 519 nm, respectively, for the TUNEL assay and 350 nm (excitation) and 461 nm (emission) for Hoechst 33342. We further analyzed the images using ImageJ software.

#### 2.8.4 Statistical Analysis

The statistical differences among data groups were analyzed using two-way analysis of variance with Tukey’s post hoc test. In all of the comparisons, a *p*-value of 0.05 or lower was considered significant. All the analyses were performed using GraphPad Prism Software version 9.3.1.

## 3 Results

### 3.1 *Candida* Species Biofilm Biomass and Metabolic Viability Quantification by Crystal Violet Staining and XTT Method after Antifungal Lipopeptide Exposure

The lipopeptides AF_4_ and AF_5_ affect different phases of *Candida* mixed biofilms was investigated with different concentrations and also the synergetic effect was investigated with conventional antifungal drug fluconazole by quantifying biofilm biomass and metabolic activity reduction upon treatment, as shown in Figure 1.

**Figure 1:**
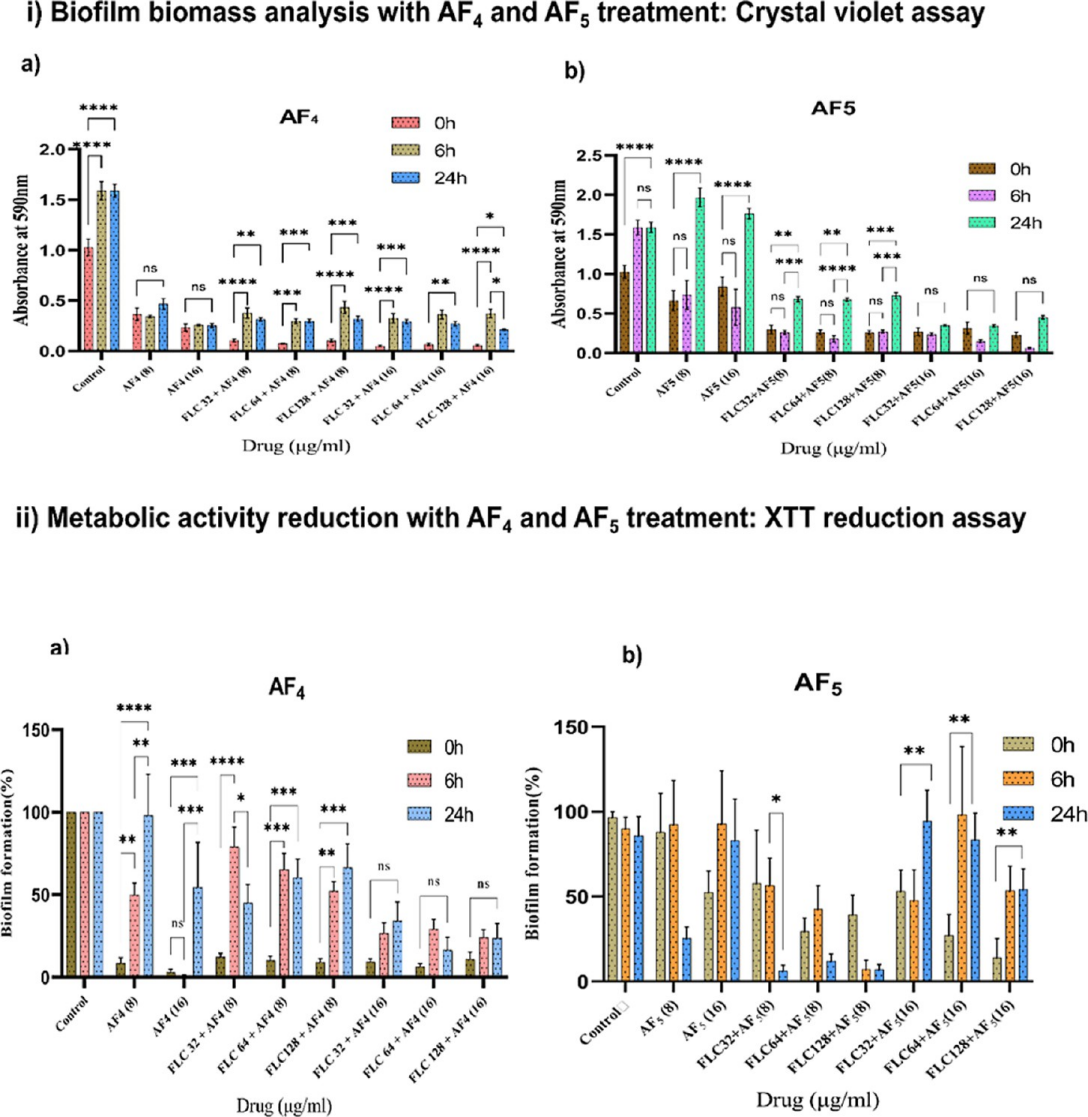
Effects of antifungal lipopeptides AF_4_ and AF_5_ on the metabolic activity and biomass of *C. glabrata* and *C. tropicalis* mixed-species biofilms at different phases. (i) Determination of the biofilm biomass. (a) Effect of AF_4_ alone or in combination with FLC on biofilm biomass. (b) Effect of AF_5_ alone or in combination with FLC on biofilm biomass. (ii) XTT metabolic activity quantification results of mixed-species biofilms. (a) Effect of AF_4_ alone or in combination with FLC on biofilm cells metabolic activity. (b) Effect of AF_5_ alone or in combination with FLC on biofilm cells metabolic activity. Treated sample values were normalized to the control, considered as (100%). Data are represented as mean ± standard error (SE) (*n* = 6) with the error bars. Statistical significance between groups is indicated in the figure (*p < 0.01, **p < 0.001, ****p < 0.0001; ns, not significant).

The biofilm biomass and metabolic activity of viable cells within the biofilm were quantified through the XTT reduction assays and the CV assays with a multiplate reader. To evaluate the prevention of adhesion, antifungal compounds were added at the beginning of the experiment to prevent in vitro adherence of log-phase mixed-species cells to the surface of PS 96-well plates, followed by a 48 h incubation for biofilm formation. *Candida* dual-species mixed biofilms were treated with AF_4_ alone at 8 and 16 μg/mL, biomass formation was reduced to 39.02% for 8 μg/mL and 24.59% for 16 μg/mL, with significant reductions in biomass (60.97% and 75.41%) respectively, and significant reductions (*P* < 0.05) in metabolic viability were (86.99%, and 92.73%) recorded. FLC at concentrations of 32, 64, and 128 μg/mL alone showed reduction neither in biomass nor reduction in metabolic activity for 0 h (Supplementary Figure 1).

However, when FLC was combined with AF_4_, the biomass formation was found to be reduced up to an average of less than 10% with significant reductions (*P* < 0.05) in biomass up to 93.1% and metabolic viability 89.57%, as illustrated in Figure 1. In contrast, AF_5_ at 8 and 16 μg/mL when used alone caused less than a 35% reduction in both biomass and viability, as determined by the XTT dye reduction assays. When combined with FLC, AF_5_ exhibited up to a 77.08% reduction in biomass and up to a 55.74% reduction in metabolic activity in a concentration-dependent manner.

For the evaluation of the antibiofilm efficacy of antifungal lipopeptides AF_4_ and AF_5_ against 6 and 24 h preformed biofilms, results are presented in Figure 1. The quantification assays clearly demonstrated that AF_4_ alone showed a significant disruption on the mature biofilms and inhibited the development of mixed biofilms. When FLC was combined with AF_4_ at 8 and 16 μg/mL, the biofilm metabolic activity was significantly (*P* < 0.05) reduced by 49.62% to 75.06% and biomass reduction was found to be above 90% for 6 h biofilms. In contrast, AF_4_ alone at a higher concentration of 16 μg/mL showed a 93.76% viability reduction (*P* < 0.05) and a 92.54% biomass reduction for 6 h biofilms, Figure 1.

While AF_5_ alone exhibited only 30% reduction in both biomass and viability, the combined effect improved the biomass reduction to 82.72% and 57.15% (*P* < 0.05) metabolic activity. For 24 h mixed-species biofilms, AF_4_ at 8 μg/mL alone had shown only a 20.76% viability reduction, whereas the reduction was improved up to 59.3% with combination with FLC with the same concentration AF_4_ at 8 μg/mL. However, AF_4_ at 16 μg/mL showed greater than 50% reduction in metabolic activity alone and combining with the FLC at various concentrations (Figure 1). In biomass quantification results, AF_4_ alone inhibited the biofilm biomass by up to 84.02%. The lipopeptide AF_5_ demonstrated around a 30% reduction in both biomass and viability during the developmental phase and no effect on mature biofilms similar to the standard antifungal FLC; a significant synergistic effect between AF_5_ and FLC was exhibited in both the developmental and mature phases, as shown in Figure 1.

### 3.2 SEM of Antifungal Lipopeptides’ Effect on *Candida* Mixed-Species Biofilms

FE-SEM analysis of both 6 and 24 h biofilms revealed the predominance of *C. glabrata* biofilms formed over *C. tropicalis* due to the less filamentous hyphae structure in mixed-species biofilm micrographs Figure 2. The growth of colonies on the Hi-CHROMagar differential agar surface confirmed the findings. The SEM analysis further demonstrated that mixed species formed an extensive mat-like biofilm at 24 h, marked by significant EPS production Figure 2. Treatments with 8 and 16 μg/mL resulted in no filamentous structures, with only scanty yeast cells. The 6 h biofilms comprised distinct microcolonies of intact filamentous hyphae. However, 24 h mature biofilms appeared as a tightly packed structure, with densely clustered cells forming a robust network of coaggregated EPS, as revealed in Figure 2.

**Figure 2:**
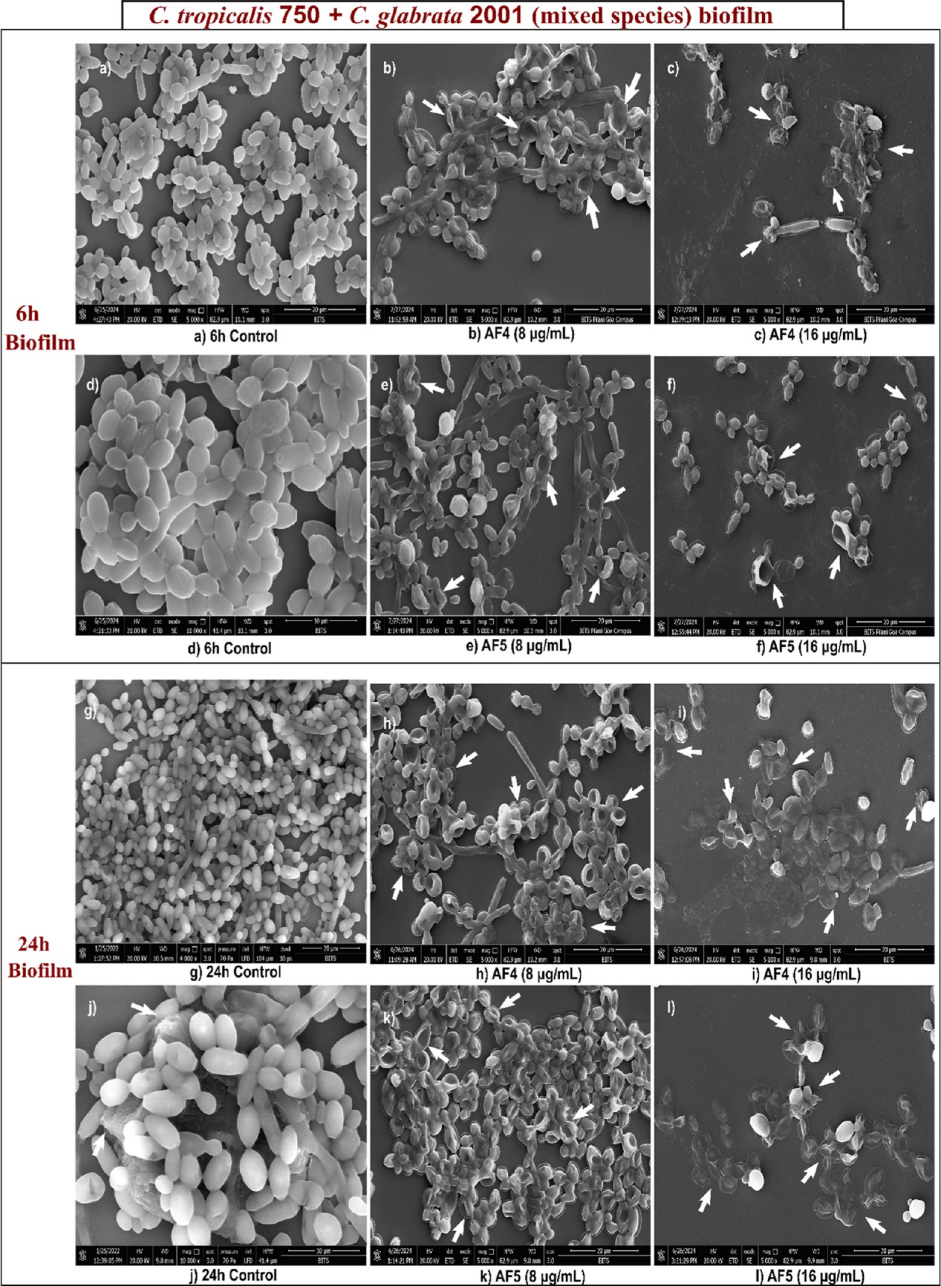
SEM of the mixed-species biofilms of *C. glabrata* and *C. tropicalis* separately treated with AF_4_ and AF_5_ at different stages of 6 and 24 h biofilms. Scale bars are at 20 μm.

Treatment with AF_4_ and AF_5_ induced significant morphological alterations in the biofilm architecture, which is characterized by rough and distorted cell surfaces and disturbed outer membranes. In contrast, untreated yeast cells exhibited intact structures with well-defined, smooth surfaces. At a concentration of 16 μg/mL, the AF_4_ lipopeptide greatly reduced the number of cells and damaged or disrupted biofilms, Figure 2. Similarly, the AF_5_ lipopeptide at 16 μg/mL caused significant damage to biofilm clusters, weakening their structure, as noticed from the disrupted or deformed cell surfaces as shown in Figure 2. Although cell shrinkage was rare in yeast morphology, substantial damage and rupture of biofilm cell surfaces were clearly visible.

### 3.3 CSLM Analysis of Antifungal Lipopeptides’ Effect on *Candida* Mixed-Species Biofilms

Confocal microscopy analysis of *Candida* mixed (dual-species) biofilms exhibited a predominance of *C. glabrata* over *C. tropicalis* biofilms, as correlated with SEM results. Confocal microscopy visualization demonstrated the strong antibiofilm properties of the investigated lipopeptides at both concentrations (8 and 16 μg/mL) compared to the 24 h control biofilms.

The number of oval-shaped spherical *C. glabrata* yeast cells in biofilm was evident; cylindrical hyphae structures of *C. tropicalis* were not observed in 6 h biofilms Figure 3. However, many metabolically active cells in 6 h biofilms were observed. It was observed that biofilms not exposed to any drugs showed a densely packed green mat-like structure, which showed a copious amount of EPS production in 24 h biofilms. This was visualized by ConA binding, as depicted in Figure 3. The cells metabolized FUN-1, which made the appearance of reddish fluorescence, and the ConA binding to α-mannopyranosyl and α-glucopyranosyl residues present in polysaccharides of yeast cell walls^37^ exhibited greenish fluorescence. AF_4_ at 8 and 16 μg/mL and AF_5_ at 16 μg/mL showed scanty living cells or small clusters where most of the cells were either not metabolically active or embedded in the biofilm matrix. However, at both concentrations, the biofilm disruptions were highly evident.

**Figure 3:**
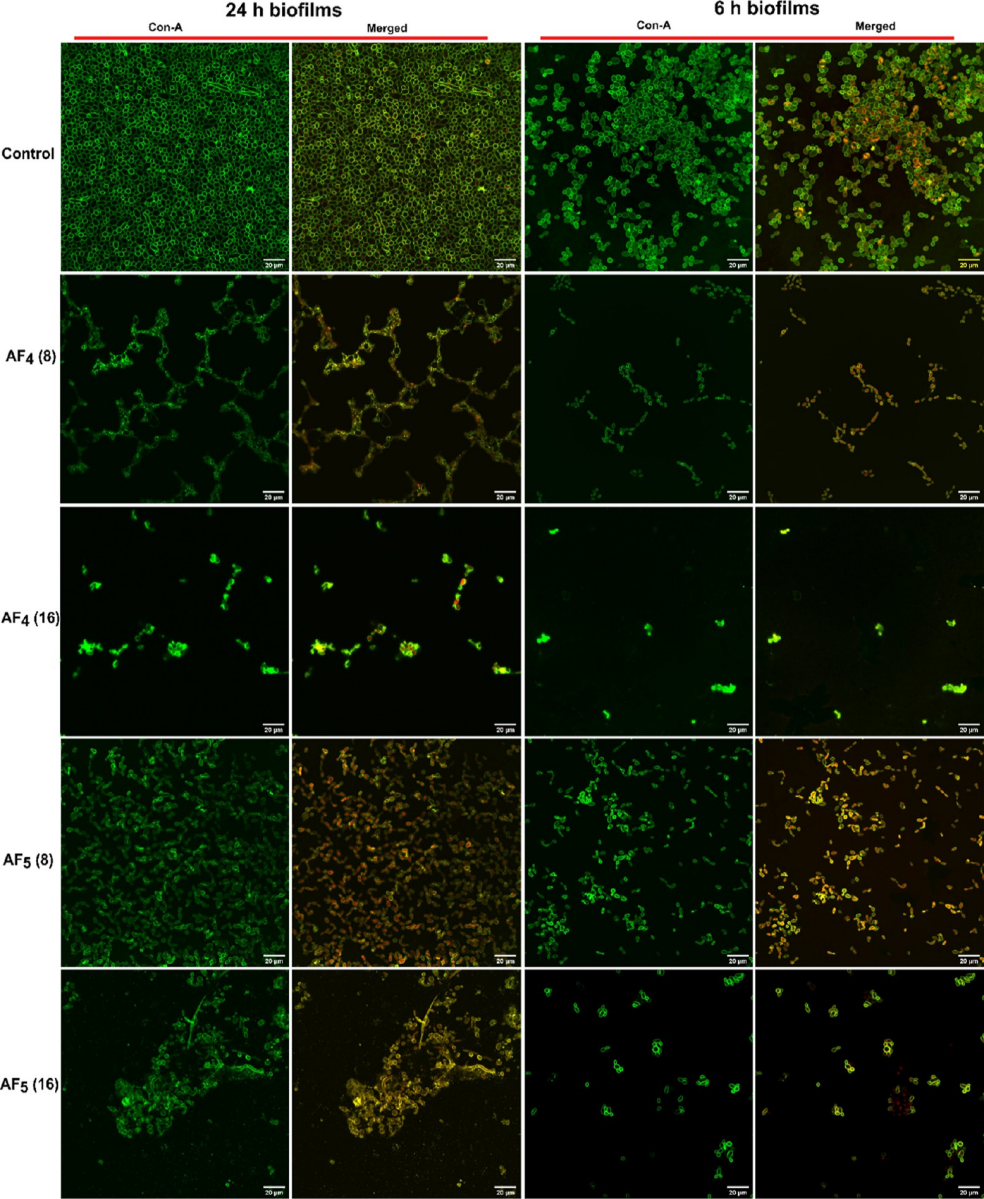
Morphological CSLM analysis of the mixed-species biofilms treated with AF_4_ and AF_5_ on different stages of 6 and 24 h biofilms. Images were acquired using the confocal scanning laser microscope at a 60× oil immersion objective with a scale bar of 20 μm.

### 3.4 Estimation of EPS Contents after Antifungal Lipopeptide Treatments

Polysaccharides are essential components of the EPS within *Candida* dual-species mixed biofilms, playing a pivotal role in maintaining their structural integrity and functionality. The phenol-sulfuric acid method was employed to quantify EPS, providing a comprehensive analysis of the effects of the antifungal agents AF_4_ and AF_5_ on the EPS layer of *Candida* mixed-species biofilms during various stages including adhesion, development, and maturation. The results, presented in Figure 4A, revealed a significant reduction in EPS percentages as compared to the controls. Biofilms treated by AF_4_ concentrations at 8 and 16 μg/mL resulted in a significant reduction in the EPS content compared to AF_5_-treated biofilms. Interestingly, these observations were found to be similar in all three stages of biofilms. However, compared to the untreated control groups and FLC-treated biofilms, AF_4_ and AF_5_-treated samples demonstrated a significant reduction in the EPS content.

**Figure 4:**
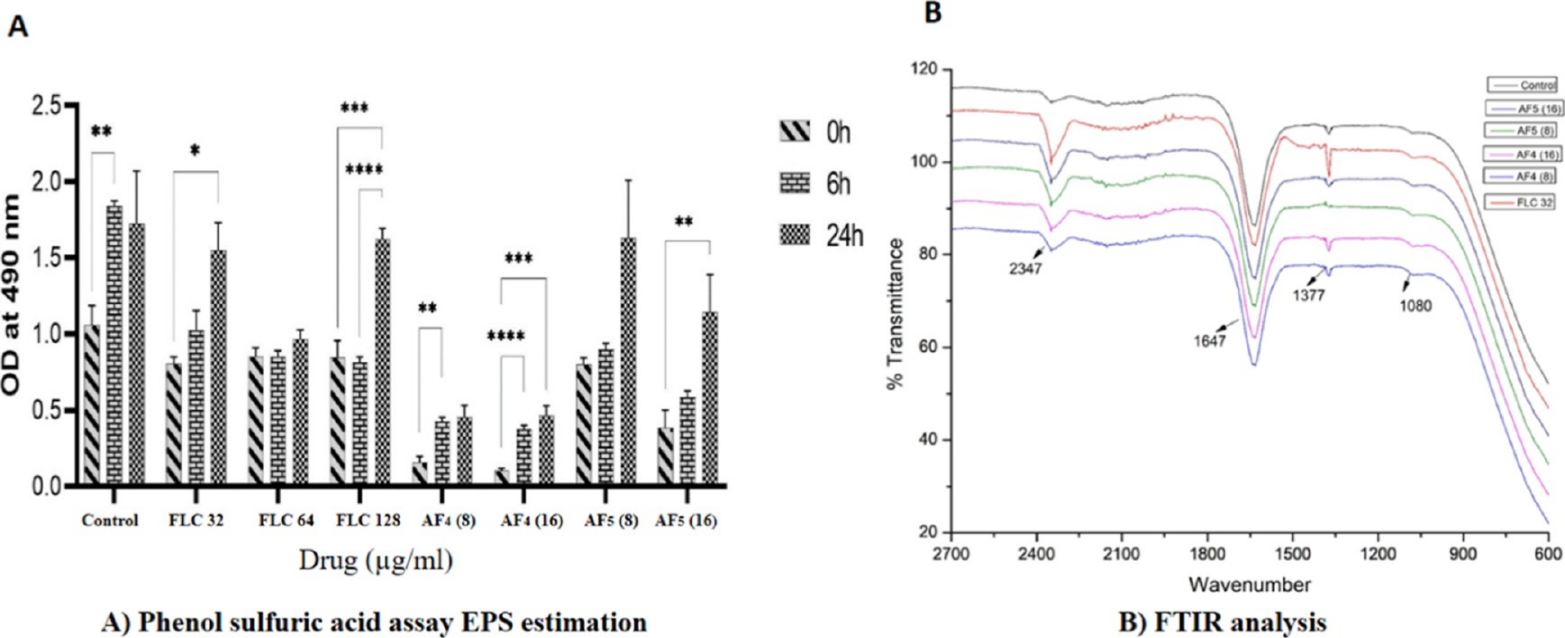
A) EPS estimation by phenol-sulfuric acid assays. *Candida* mixed-species biofilms at different stages of 0, 6, and 24 h treated with FLC, AF_4_/AF_5_ alone at different concentrations. Data are represented as mean ± SE (*n* = 6) with error bars. B) Generation of high-resolution ∼2700–600 cm^–1^ spectrum was done using the ATR-FTIR spectroscopy. Effects of FLC, AF_4_/AF_5_ on the biochemical composition of *Candida* mixed-species biofilms.

### 3.5 ATR-FTIR Analysis

Since EPS is composed of lipids, proteins, and exopolysaccharides, FTIR spectral shifts provided qualitative evidence of compositional alterations induced by antifungal lipopeptide treatments, thereby supporting the biochemical data. The ATR-FTIR method was employed to investigate the changes in biochemical nature induced by lipopeptides in 24 h *Candida* mixed-species mature biofilms. The analysis targeted the spectral region of ∼2700–600 cm^–1^, encompassing markers for key EPS components including lipids, proteins, and polysaccharides. The FTIR spectra of the biofilms showed different transmittance profiles for different functional groups in this spectral range. As shown in Figure 4B, the FTIR findings align with those reported in earlier studies.^29,30^ The main signals in the spectra were linked to functional groups related to amides, lipids, and polysaccharides. Notable peaks included amide I at 1647 cm^–1^, amide II at 1377 cm^–1^, and carboxylic acid COO^–^ symmetric stretches. These alterations were observed in the presence of AF_4_ and AF_5_ lipopeptides at 8 and 16 μg/mL concentrations on 24 h biofilms. Overall, the significant impacts of AF_4_ and AF_5_ lipopeptides on *Candida* mixed-species biofilms were evident through changes in the intensity of the ATR-FTIR fingerprint region (∼2700 to 600 cm^–1^), both with and without lipopeptide treatment, as depicted in Figure 4B.

### 3.6 Annexin V-FITC/PI Staining Results

Phosphatidylserine (PS) externalization in apoptotic cells was evaluated by flow cytometry using Annexin V-FITC and PI dual staining in *C. tropicalis* and *C. glabrata* after treatments with antifungal lipopeptides AF_4_ and AF_5_ at different concentrations. In the process of apoptosis induction with the drug treatments, the PS translocated to the outer leaflet of the plasma membrane was specifically stained by FITC-conjugated Annexin V binding, whereas PI specifically binds to nucleic acids in membrane-compromised cells as a marker for necrosis.

Two strains, including *C. tropicalis* and *C. glabrata*, were exposed to sub-MIC (2 μg/mL) and at minimum inhibitory concentration (MIC) (4 μg/mL), as well as with 2x MIC (8 μg/mL) of AF_4_ and AF_5_ lipopeptides. The inner leaflet of the cell membrane typically harbors PS. However, apoptotic cells externalize PS to the outer membrane surface as an early marker of apoptosis in fungi. For a study on antifungal-treated *Candida* cells, PS externalization by flow cytometry, Annexin V-FITC, and PI dual stains were used on *C. tropicalis* and *C. glabrata* cells treated with the new antifungal lipopeptides AF_4_ and AF_5_ at 2, 4, and 8 μg/mL for 12 h. In the process of apoptosis induction, PS moved to the outer leaflet of the plasma membrane and was stained with FITC-conjugated Annexin V binding. On the other hand, PI binds to nucleic acids in cells that have lost their membrane integrity, which is a sign of necrosis.

For the apoptosis assays, cells of two strains, such as *C. tropicalis* and *C. glabrata*, were exposed to sub-MIC, 1× MIC, and 2× MIC of AF_4_ and AF_5_ lipopeptides. A significant shift from live cells to early stage and late-stage apoptotic induction percentage was increased, along with the lipopeptides AF_4_ and AF_5_ increasing the concentration from 1× MIC to 2× MIC. In comparison, sub-MIC had more live cells, as seen in Figure 5 for *C. glabrata* ATCC 2001 and Figure 6 for ATCC 750. Quadrant 2 broadly distributed the lipopeptide-treated cells, indicating late-stage apoptosis or cell death, whereas quadrant 3 (99%) distributed no drug-treated cells that appeared negative for either strain. When lipopeptides were used at the same concentration or higher than the MIC, most cells showed signs of late apoptosis, indicated by being in quadrant 2 (Annexin V^+^ and PI^+^) leaving only a few cells in quadrant 4 (Annexin V^+^ and PI^–^). However, cells treated with greater than 4 μg/mL lipopeptides shifted toward necrosis; these were the cells in quadrant 1 (Annexin V^–^, PI^+^).

**Figure 5:**
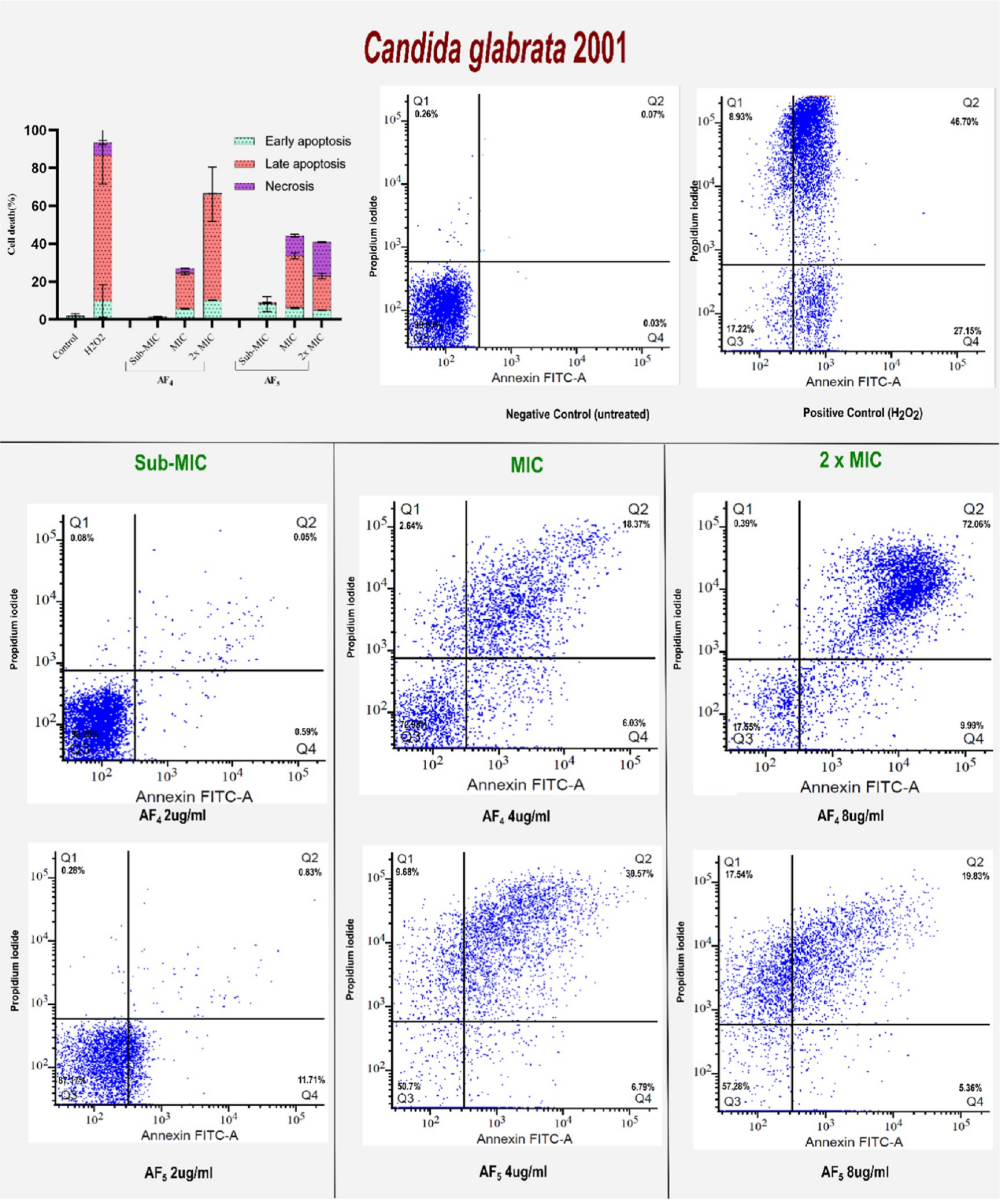
Determination of different stages of cell death of AF_4_ and AF_5_-treated *C. glabrata* ATCC 2001 cells by the AnnexinV-FITC conjugate. The cell apoptosis data are represented as quadrant dot plots, where Q_1_ is necrosis (Annexin V^–^/PI^+^), Q_2_ is late apoptosis (Annexin V^+^/PI^+^), Q_3_ is viable cells (Annexin V^−^/PI^−^) and Q_4_ is early apoptosis (Annexin V^+^/PI^−^).

**Figure 6:**
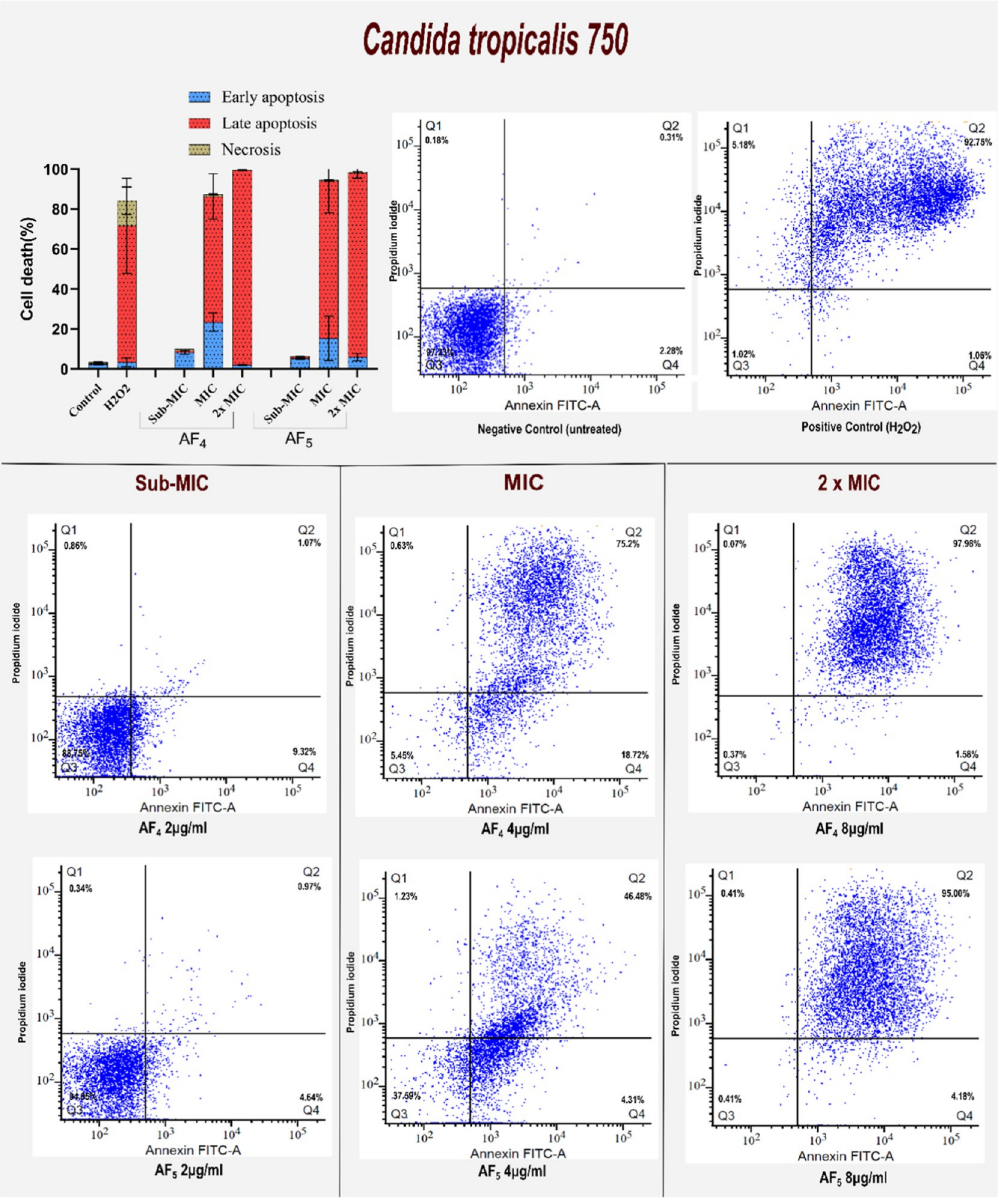
Determination of different stages of cell death of AF_4_ and AF_5_-treated *C. tropicalis* ATCC 750 cells by Annexin V-FITC conjugate. The cell apoptosis data are represented as quadrant dot plots, where Q_1_ is necrosis (Annexin V^–^/PI^+^), Q_2_ is late apoptosis (Annexin V^+^/PI^+^), Q_3_ is viable cells (Annexin V^−^/PI^−^) and Q_4_ is early apoptosis (Annexin V^+^/PI^−^).

### 3.7 TUNEL Assay

PS externalization is a key parameter in apoptosis-related studies, especially during late-stage apoptosis, when chromosome condensation and DNA damage occur. The TUNEL assay was employed to investigate the presence of late-stage apoptosis in response to the active drugs. The TUNEL assay is based on the incorporation of modified dUTP at the 3′-OH ends of fragmented DNA (Phillips et al., 2003). Staining the *Candida* cells with a Hoechst dye for differentiation produces blue fluorescence for live cells. Figure 7 illustrates that the *C. tropicalis* cells exposed to AF_4_ at 2, 4, and 8 μg/mL the lipopeptides showed a significant increase of TUNEL-positive nuclei with green fluorescence. *C. glabrata* cells exposed to the AF_4_ at 2, 4, and 8 μg/mL resulted in DNA fragmentation in a concentration-dependent manner, as illustrated in Figure 8. The effect of AF_5_ at 2, 4, and 8 μg/mL on *C. tropicalis* and *C. glabrata* cells that caused DNA fragmentation is shown in Figure 9. These findings unequivocally suggest that AF_4_/AF_5_ at 4 and 8 μg/mL concentrations can affect DNA fragmentation, as was revealed by the enhanced green fluorescence intensity in cells in TUNEL-positive nuclei.

**Figure 7:**
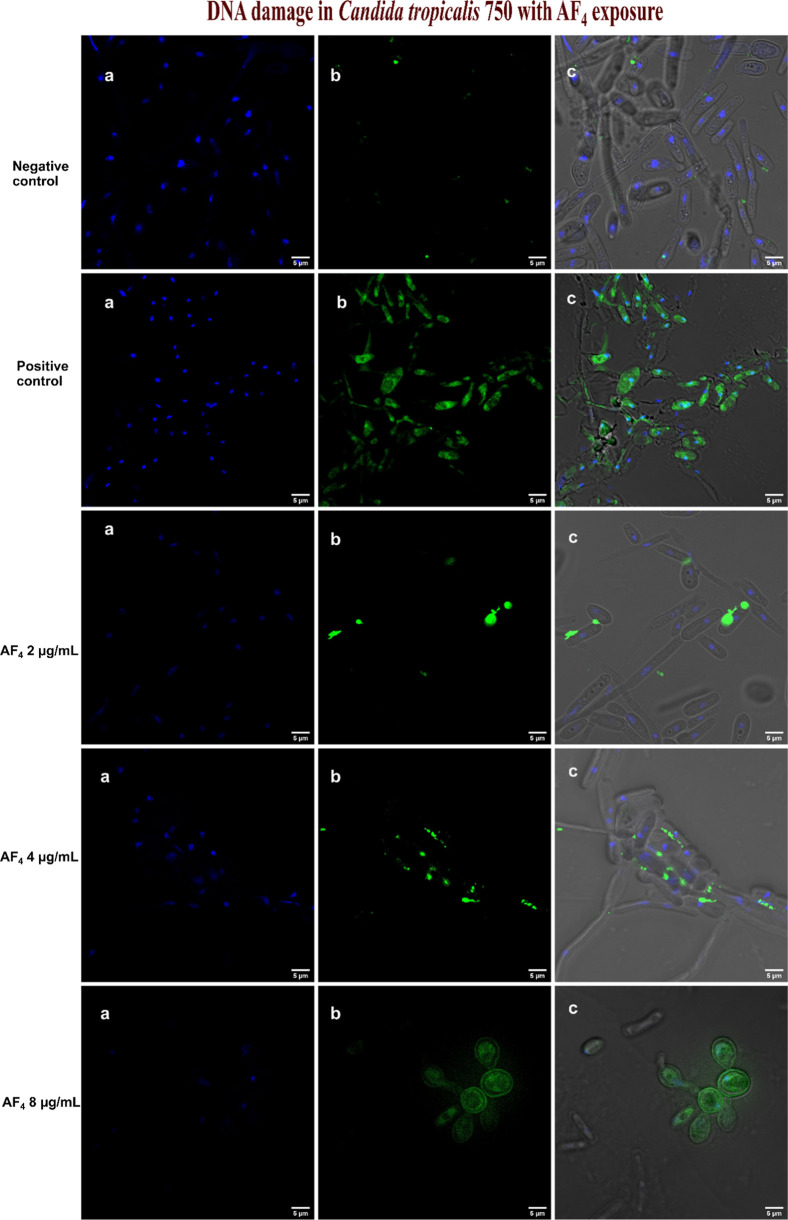
DNA damage determined by TUNEL assay in *C. tropicalis* ATCC 750 cells after exposure to (0.5, 1, and 2× MIC of AF_4_). (a–c) Cells stained with Hoechst 33342 dye (blue fluorescence for intact cells) and green fluorescence by Alexa Fluor 488, indicating DNA damage and the merged image, respectively. Scale bars: 5 μm.

**Figure 8:**
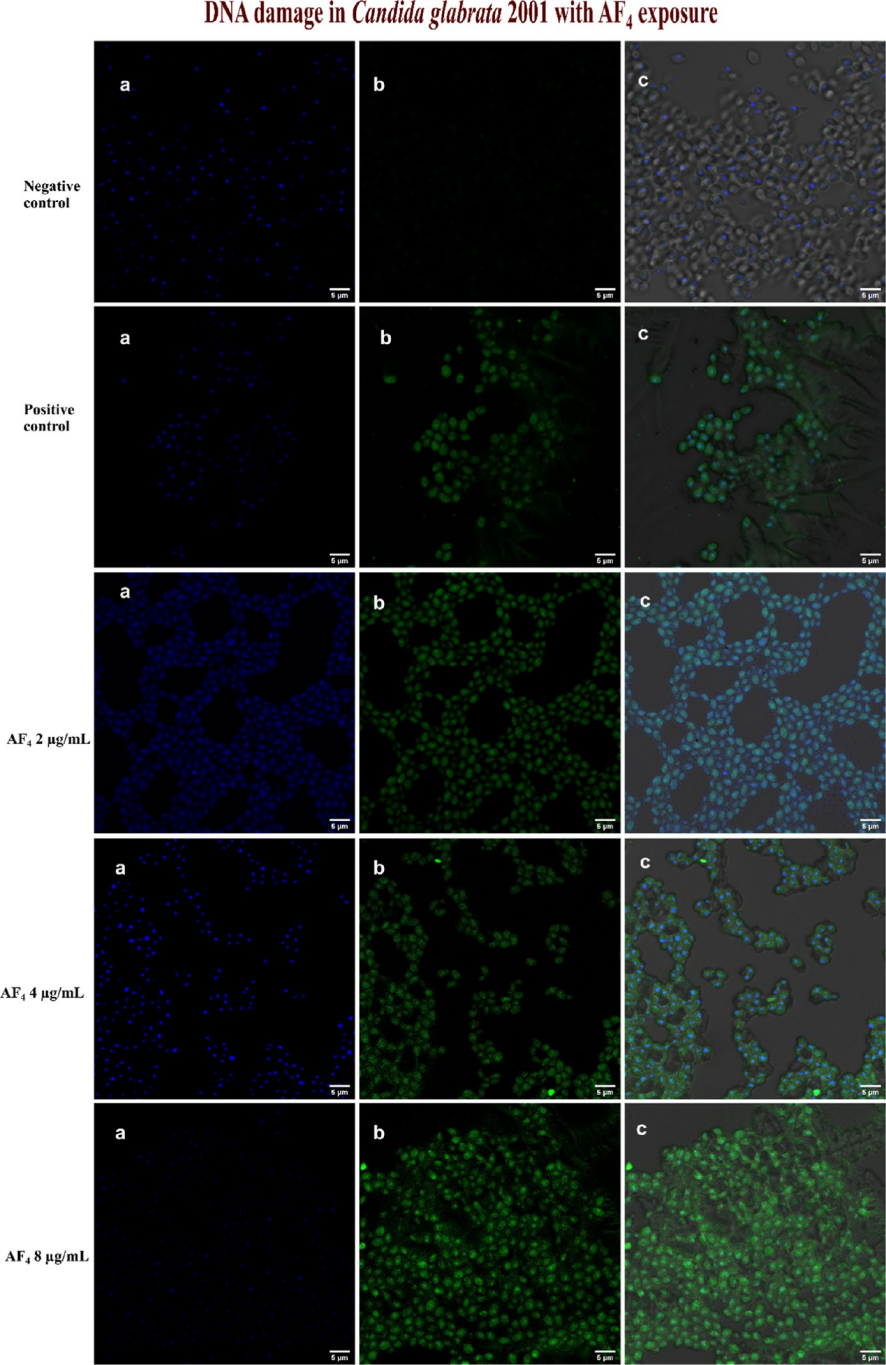
DNA damage determined by TUNEL assay in *C. glabrata* cells after exposure to (0.5, 1, and 2× MIC of AF_4_). (a–c) Cells stained with Hoechst 33342 dye (blue fluorescence for intact cells) and green fluorescence by Alexa Fluor 488, indicating DNA damage and the merged image, respectively. Scale bars: 5 μm.

**Figure 9:**
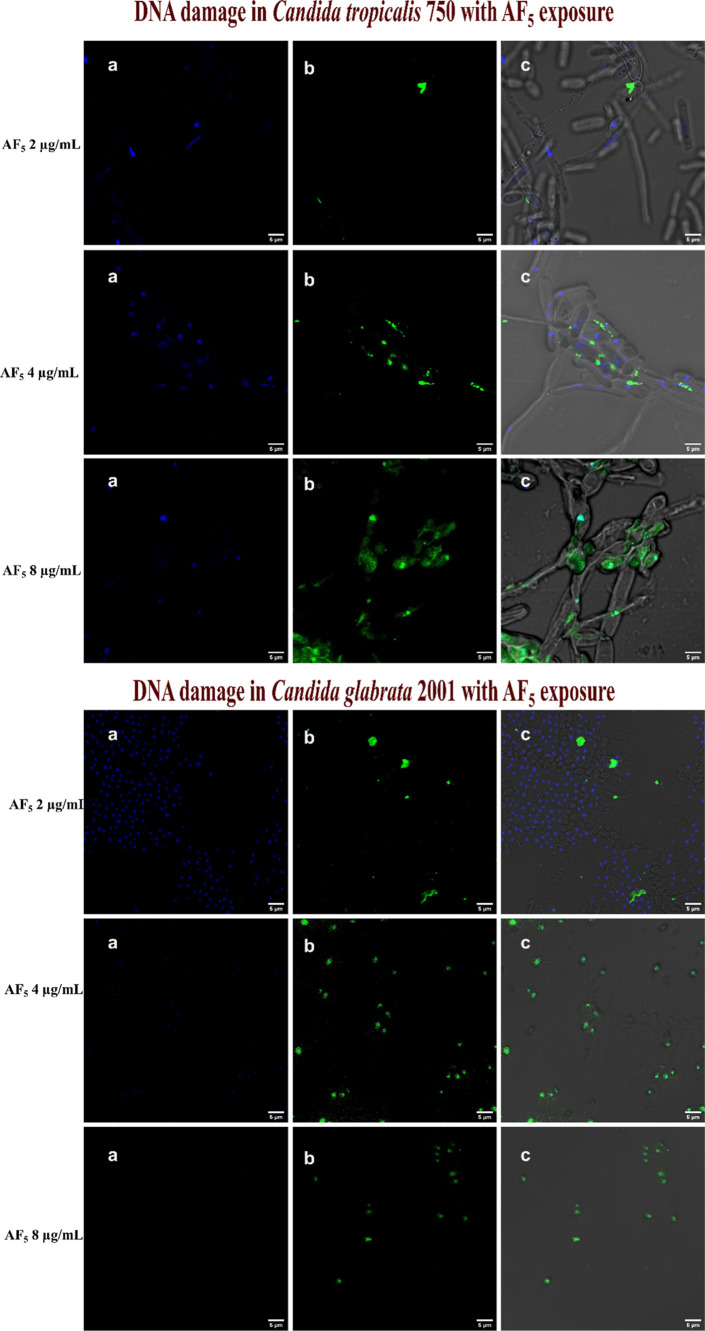
DNA damage determined by TUNEL assay in *C. glabrata* 2001 and *C. tropicalis* 750 cells after exposure to (0.5, 1, and 2× MIC of AF_5_). (a–c) Cells stained with Hoechst 33342 dye (blue fluorescence for intact cells) and green fluorescence by Alexa Fluor 488 and a merged, respectively. Scale bars: 5 μm.

For the positive control, *C. tropicalis* and *C. glabrata* cells were treated with 30 mM H_2_O_2_ and exhibited increased green fluorescence, indicating the presence of late apoptotic cells compared to the negative control, with the majority of the population belonging to the blue fluorescent live cells. The finding indicates that AF_4_ at concentrations of 4 and 8 μg/mL can impact DNA fragmentation, as shown by the increased green fluorescence in cells with TUNEL-positive nuclei.

## 4 Discussion

Polymicrobial biofilms have remained a significant concern in clinical settings as they contribute to multiple challenges, including antimicrobial treatment failure, evasion of the host immune system, development of antimicrobial resistance, and the persistence of chronic or recurrent infections.^17^ These biofilms consist of more than one microbial species interacting within a shared extracellular matrix, which enhances their resilience against treatments and environmental stressors.^38^ Fungi are endowed with the ability to aggregate at different interfaces, forming fixed communities such as single- or mixed-species fungal biofilms, demonstrating remarkable environmental adaptability.^10,39^ Comprehending the interactions between microorganisms is essential to unravel the mechanisms of their pathogenicity and could pave the way for the development of alternative therapies to manage such infections effectively.^40,41^ Adherence of sessile cells to the surface is the initial stage of biofilm formation. Increasing hydrophobicity on the surface of the material will reduce the adherence of the cells to the surface.^42^ In this study, we explored the effect of AF_4_ and AF_5_ on *Candida* mixed biofilms. This study aimed to investigate the impact of antifungal compounds on *Candida* biofilm formation and maturation using the CV assay.^32^ We examined the biofilm biomass at three key stages: adherence (0 h), development (6 h), and maturation (24 h) in both treated and nontreated conditions. Surface adherence, proliferation, or developmental and maturation, followed by the dispersion phase, are the distinct stages of biofilm formation cycles.^27,43,44^ During 6 h, the early phase of biofilm development is characterized by microcolony formation with the upregulation of efflux pumps, which are often associated with drug resistance. Generally, the early biofilm stage remains susceptible to antimicrobial drugs due to their lower cell density and lack of EPS matrix.^45–47^ The investigating lipopeptides AF_4_ and AF_5_ exhibited strong antibiofilm efficacy by inhibiting both adherence and proliferation. *Candida* biofilms are highly resilient to treatment due to the physical barrier provided by the biofilm matrix.^48^ Mature biofilms (24 h) consist of microbial cells embedded within a well-developed EPS matrix and display elevated antifungal resistance or reduced susceptibility to antifungals.^49–51^ Results revealed that AF_4_ and AF_5_ not only prevented the adhesion of two mixed *Candida* species to surfaces but also disrupted preformed biofilms at both 6 and 24 h. A comprehensive understanding of the biofilm architecture could be made through advanced microscopic analysis (SEM and CSLM), which supported the quantitative assay results. Biofilm quantification methods, including XTT metabolic activity assays and CV assays for biomass, revealed a significant reduction of more than 90% in both metabolic activity and biomass at 8 and 16 μg/mL concentrations of AF_4_ compared with the untreated controls. In contrast, AF_5_ alone resulted in only about a 30% reduction. Consistent with these findings, advanced microscopy demonstrated clear structural alterations in AF_4_/AF_5_-treated biofilms, including reduced cell density, loss of the EPS matrix, damaged hyphal structures, and concentration-dependent morphological changes such as cell wall shrinkage or swelling, separation of the outer cell wall layer, and accumulation of hyphal debris. The observations suggest the lipopeptides antibiofilm effect. The production of biofilms by *Candida* species is a significant contributor to their pathogenicity as it enhances their resistance to antimicrobial agents and host immune responses. These studies might indicate complex regulatory networks involving numerous genes and pathways that are crucial for elucidating the molecular mechanisms in other fungal species. The previous studies^52,53^ provided valuable insights into these microbial interactions. Moreover, *C. glabrata* seems to interfere with or inhibit the mechanisms responsible for the adherence of *C. albicans* in mixed-species biofilms.^21^ This competitive interaction likely reduces the pathogenic potential of *C. albicans*, highlighting the complex dynamics of microbial cohabitation within biofilms.

Interestingly, further studies revealed that these interactions can vary depending on the environmental conditions. For instance, dual-species biofilms of *C. albicans* and *C. glabrata* displayed a significantly higher total biomass compared to monospecies biofilms of either yeast cells. In a previous study,^15^ the interactions were explored and it was noted that factors such as strain characteristics, culture composition, and sugar replacements significantly influenced biofilm formation and biomass production. The study suggests that interactions between *Candida* species might be highly dependent on many variables, indicating that the biofilm’s characteristics are not only species-specific but also influenced by external conditions and nutrient availability.^54^

Combining FLC with lipopeptides (AF_4_/AF_5_) demonstrated a significant inhibitory effect on developing and mature biofilms compared to FLC alone. The synergistic interactions between these lipopeptides (AF_4_/AF_5_) and FLC were determined through a checkerboard assay and evaluated using *C. glabrata* planktonic cells, similar to our previous observations against *C. tropicalis* ATCC 750 ^23^. The amphiphilic nature of lipopeptides AF_4_/AF_5_, due to their peptide sequence (*Asn-Pro-Tyr-Asn-Gln-Thr-Ser*) combined with a hydrophobic fatty acid tail (the alkyl lipid moiety length^26^ varies between homologous lipopeptides AF_4_ and AF_5_), enables their interaction with *Candida* cell membranes. This interaction may occur through penetrating or altering cell membrane, potentially forming dents or pores that might lead to the leakage of contents, or via diverse mechanisms such as forming a complex of membrane ergosterol-lipid moiety, membrane perturbation (as was revealed by the series of the series of 1,6-diphenyl-1,3,5-hexatriene (DPH) assays assays from our laboratory), cell surface alterations, and targeting cellular processes including oxidative stress, reactive oxygen species (ROS) generation, and apoptosis.^24,55^ Thus, lipopeptides have independent antifungal mechanisms beyond biofilm disruption. Exposure of *C. tropicalis* and *C. glabrata* planktonic cells to increasing concentrations of AF_4_ and AF_5_ produced a clear dose-dependent induction of apoptosis, as evidenced by multiple hallmark markers. FITC Annexin V/PI staining revealed that at MICs, both AF_4_ and AF_5_ predominantly induced late-stage apoptosis (PS externalization without loss of membrane integrity), whereas at 2× MIC concentrations, a shift toward necrosis was observed. A FITC-dUTP nick-end labeling (TUNEL) assay was conducted to detect DNA fragmentation associated with apoptosis in yeast cells treated with AF_4_ and AF_5_ lipopeptides. Figures 7–9 show that there was hardly any detectable signal in the untreated cells; however, green fluorescence was clearly observed in the treated cells, suggesting that the lipopeptides induced apoptosis in treated yeast cells. DNA fragmentation, a hallmark of late-stage apoptosis, aligns with previous reports of small-molecule-induced programmed cell death in *Candida* spp. The TUNEL assay results parallel the Annexin V-FITC/PI assay findings, showing a concentration-dependent increase in the number of TUNEL-positive nuclei, indicating progressive DNA fragmentation in a dose-dependent manner. Additional mechanistic assays in our previous studies demonstrated concomitant ROS generation and DNA damage, suggesting that AF_4_ and AF_5_ may initiate apoptosis through an oxidative stress-mediated process, culminating in DNA fragmentation.^22,55^ ROS generation is a key contributor to antifungal activity and is often associated with early apoptosis. Using the fluorescent dye 2′,7′-dichlorodihydrofluorescein diacetate (DCFH-DA), we previously determined ROS accumulation in lipopeptide (AF_4_)-treated *C. tropicalis* ATCC 750 (Swetha et al., 2023)^56^ and *C. glabrata* ATCC 2001 cells.^22^ Furthermore, the effect of lipopeptides on the mitochondrial membrane potential was inferred from the accumulation of rhodamine 123 within the mitochondrial membrane, as demonstrated in our earlier work (Swetha et al., 2023). ROS generation, phosphatidylserine (PS) externalization, and DNA fragmentation have been reported to be observed in fungal cells undergoing apoptosis.^55–57^

The synergistic effect of these iturinic compounds with surfactant properties plays a key role in FLC penetration through the membrane damage or alters the mixed-species EPS production or disruption that might turn to improve the accessibility of FLC to its intracellular target to inhibit more effectively the lanosterol 14α-demethylase, which is a key enzyme in the ergosterol biosynthesis pathway.^58^ The findings derived from this study underscore the importance of exploring the antibiofilm potential of antifungal lipopeptides against microbial interactions within mixed biofilms to identify novel therapeutic strategies. The ability of *C. glabrata* to inhibit or suppress the growth of other *Candida* species, like *C. albicans* or *C. tropicalis*, highlights the competitive dynamics within biofilms.^15^ The promising antibiofilm activities of AF_4_ and AF_5_ highlight their potential application in hydrogel-based delivery systems. Short battacin-based lipopeptides that constitute self-assembling antimicrobial hydrogels with activity against *Pseudomonas aeruginosa* and *Staphylococcus aureus* have been reported recently.^59^ Hydrogels provide a moist, biocompatible, and controlled-release platform that can localize lipopeptides at the site of infection, thereby enhancing therapeutic efficacy and minimizing systemic toxicity. Incorporation of lipopeptides into hydrogels could be particularly valuable for the treatment of *Candida*-associated mucosal infections, wound biofilms, and device-related infections, where biofilm persistence poses a major clinical challenge. Moreover, hydrogel formulations are versatile, allowing for topical, injectable, or coating-based applications, thereby broadening their translational scope from wound dressings and vaginal gels to catheter coatings and implant surfaces.^60^ Meanwhile, the efficacy of antifungal lipopeptides such as AF_4_ and AF_5_ can potentially open new avenues for targeted treatments to combat these complex and resilient biofilm-associated infections.

## 5 Conclusion

The overall findings described here indicate that the antifungal lipopeptides AF_4_ and AF_5_ have multipronged modes of action that cause PS externalization and DNA degradation. These lipopeptides likely disrupted the biofilm’s architecture or inhibited microbial growth within it, demonstrating their potential as effective antibiofilm agents. The prevention of biofilm formation and the eradication of mature biofilms in the clinical settings always present formidable challenges. Comprehending the effects of the novel investigational lipopeptides on the clinically relevant mixed-species biofilms may assist in overcoming the challenges of the existing antibiofilm strategies.

## Supplementary Material



## Notes

Effects of antifungal lipopeptides fluconazole (FLC) on the metabolic activity and biomass of *C. glabrata* and *C. tropicalis* mixed-species biofilms; determination of the biofilm biomass quantification; and XTT metabolic activity quantification results of mixed-species biofilms (PDF)
