# Supplementary material for: Unveiling the In Vitro Antibiofilm Efficacy of Antifungal Lipopeptides Purified from Bacillus sp. against Mixed-Species Biofilms of Candida
Source: ACS Omega. 2025 Nov 21;10(48):58671–87. doi: 10.1021/acsomega.5c06938 (PMC12771234; doi:10.1021/acsomega.5c06938)
Supplement: Supplementary file 1 [file ao5c06938_si_001.pdf]

Supplementary File

Unveiling the in vitro antibiofilm efficacy of antifungal lipopeptides purified from *Bacillus* sp. against mixed-species biofilms of *Candida*

Madhuri Madduri[a,] Shivaprakash M. Rudramurthy[b], *Utpal Roy[a]

Affiliations:

[a,] Department of Biological Sciences, BITS Pilani K.K. Birla Goa Campus, NH 17B Bypass Road, Goa 403726, India

[b,] Department of Medical Microbiology, Post Graduate Institute of Medical Education and Research (PGIMER), Chandigarh 160012, India

***Corresponding author:** Utpal Roy, [c]Department of Biological Sciences, BITS Pilani K.K. Birla Goa Campus, NH 17B Bypass Road, Goa 403726, India. Email**:** utpalroy@gmail.com

Figure S1.

A)

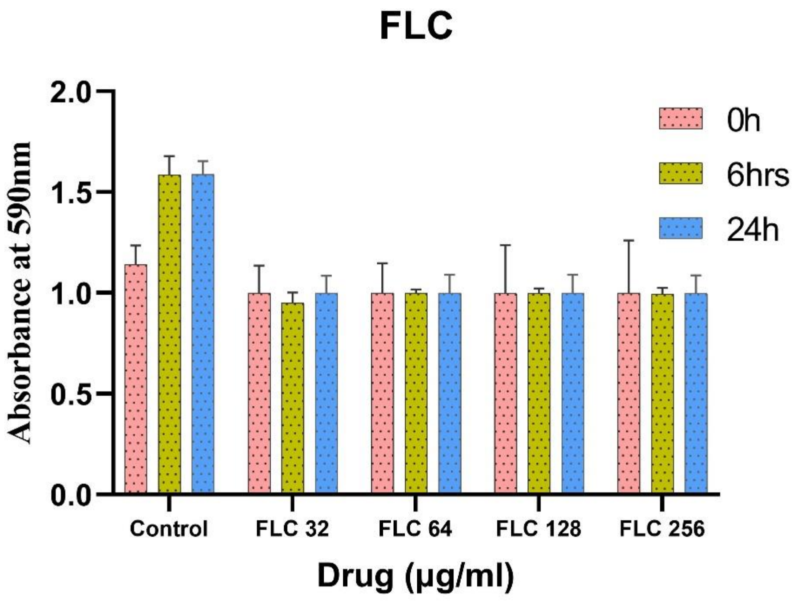

B)

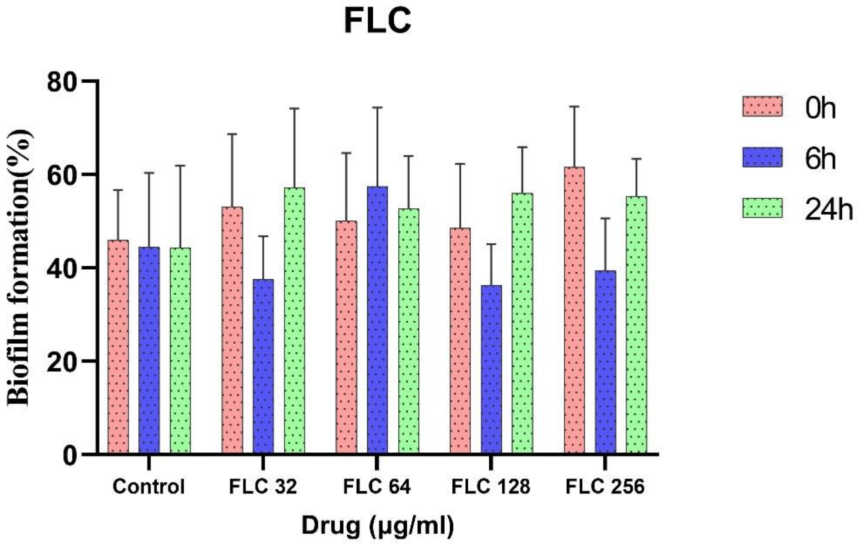

Figure S1. Effects of FLC at varying concentrations on the biomass and metabolic activity of *C. glabrata* and *C. tropicalis* mixed-species biofilms (a) Determination of the biofilm biomass quantification. (b) XTT metabolic activity quantification results of mixed-species biofilms. Treated sample values were normalized with control, considered as (100%). Data are represented as mean ± SE (n = 6) with the error bars.
